# Development of depressive symptoms post hip fracture is associated with altered immunosuppressive phenotype in regulatory T and B lymphocytes

**DOI:** 10.1007/s10522-015-9587-7

**Published:** 2015-06-26

**Authors:** Niharika Arora Duggal, Jane Upton, Anna C. Phillips, Janet M. Lord

**Affiliations:** School of Immunity and Infection, University of Birmingham, Birmingham, B15 2TT UK; NIHR Surgical Reconstruction and Microbiology Research Centre, Birmingham, UK; School of Sport and Exercise Sciences, University of Birmingham, Birmingham, B15 2TT UK

**Keywords:** Hip fracture, Depression, Regulatory T cell, Regulatory B cell, Stress, Immunesenescence

## Abstract

Hip fracture is a common physical trauma in older adults that is also associated with a high incidence of new onset depression. The immune system declines with age and is also compromised by physical and psychological stress. This study examined whether hip fracture and depressive symptoms had additive effects upon the aged immune system that might contribute to poor health outcomes after hip fracture. We assessed the frequency of regulatory T cells, T_regs_ (CD4^+^ CD25^+^ Foxp3^+^) and IL10 production by CD4 T cells, and the frequency and IL10 production by regulatory B cells, B_regs_ (CD19^+^ CD24^hi^ CD38^hi^) in 101 hip fracture patients (81 female) 6 weeks after injury and 43 healthy age-matched controls (28 female). 38 hip fracture patients (37 %) developed depressive symptoms. Hip fracture did not have an effect on circulating T_regs_ frequency but a significant reduction in the frequency of B_regs_ was observed in patients who developed depression compared with non-depressed patients (p = 0.001) or healthy controls (p < 0.001). B_regs_ also showed a significant decline in IL10 production in depressed hip fracture patients compared with controls (p = 0.04) and non-depressed patients (p = 0.01). In contrast, there was an increase in IL10 production by CD4 T cells in hip fracture patients with new onset depression compared to hip fracture patients without depression (p = .04) and healthy controls (p = .02). We conclude that the reduced immunity associated with new onset depression post hip fracture could include a contribution by heightened T_regs_ function.

## Introduction

Hip fracture is a common and devastating injury and a major health issue in old age (Abrahamsen et al. 2009). 1 in 3 adults aged 65 years and over fall each year and in the UK alone this results in approximately 75,000 hip fractures each year. Hip fracture is a frequent cause of institutionalisation and has a 1 year mortality of approximately 25 % (Panula et al. 2011) with infections a major cause of morbidity and mortality in this patient group (Edwards et al. 2008). The reasons for such poor outcomes in this trauma population are multifactorial but include co-morbidities (Edwards et al. 2008; Panula et al. 2011), reduced immunity (Butcher et al. 2005) and the consequences of depression (Phillips et al. 2013). Healthy older individuals have been reported to experience greater levels of stress, anxiousness and depression than young adults (Luz et al. 2003). Stressful life events such as bereavement or a disabling medical event are amongst the most potent factors that can trigger depressive symptoms (Cole and Dendukuri 2003). Therefore, it is perhaps not surprising that a high rate of depression (9–47 %) has been reported in studies of older adults with hip fracture (Holmes and House 2000). Importantly, depression in hip fracture patients has been associated with increased risk of infections and poor survival (Nightingale et al. 2001), impaired recovery and a retarded ability to regain pre-fracture levels of physical functioning (Mossey et al. 1990).

It is well documented that ageing is accompanied by immune decline, termed immunesenescence, which contributes to increased susceptibility to infections in older adults (Dorshkind et al. 2009; Panda et al. 2009). However the immune system does not act in isolation and is profoundly affected by both physical and psychological stress (Butcher and Lord 2004; Segerstrom and Miller 2004). Importantly, there is accumulating evidence suggesting that the effects of stress and age are interactive, with chronic stress exacerbating the effects of ageing in older adults (Kiecolt-Glaser and Glaser 1999). In this study we set out to test the hypothesis that psychological stress, specifically depressive symptoms, would act additively with the physical stress of hip fracture to amplify the effect of ageing upon immunity. We have reported previously that after hip fracture the function of innate immune cells (neutrophils, NK cells and monocytes) was significantly suppressed but only in the patients who developed depression (Duggal et al. 2013a, 2014, 2015). Here we extend the investigation to consider the regulatory cells of the immune system that are central to immune homeostasis, in order to determine if over activity in this arm of the immune response to injury could account for reduced immunity and increased susceptibility to infections after hip fracture.

Once activated it is essential to modulate the immune response to prevent unintended harmful effects arising from the defensive actions of immune cells. Regulatory CD4^+^CD25^+^Foxp3^+^ T cells play a pivotal role in maintaining immune homeostasis by suppressing immune responses (Wing and Sakaguchi 2010). T_regs_ suppressive function is mediated via production of immunosuppressive cytokines, such as IL10 (Hara et al. 2001), TGFβ (Oida et al. 2003) and via cell–cell contact (Stephens et al. 2001). An age associated increase in the frequency of T_regs_ has been reported in mice (Kozlowska et al. 2007; Zhao et al. 2007) and humans (Gregg et al. 2005). However, concomitant impairment in the immunosuppressive function of CD4^+^ T_regs_ has been shown in aged mice (Zhao et al. 2007) and humans (Tsaknaridis et al. 2003), though the literature is inconsistent with other studies reporting intact T_regs_ suppressive function with age (Gregg et al. 2005; Lages et al. 2008). More recently, a subset of B cells has been shown to be able to suppress inflammation (Wolf et al. 1996). In humans, CD19^+^ CD24^hi^ CD38^hi^ immature transitional B cells exhibit immunosuppressive properties, mainly via IL10 production on stimulation via CD40 or the TLR pathway (Blair et al. 2010; Duggal et al. 2013b). These IL10 producing B cells restrain inflammatory Th1 and Th17 cells and maintain T_reg_ numbers (Carter et al. 2012; 2011). Tedder and co-workers have also reported the existence of a CD19^+^ CD5^+^ CD1d^hi^ B cell subset, designated as B10 cells that can exert immunosuppressive effects in a IL10 dependent manner (Yanaba et al. 2008). Recently on investigating regulatory function in healthy older adults we reported both numerical and functional impairments (reduced IL10 production) in CD19^+^ CD24^hi^ CD38^hi^ B_regs_ with advancing age (Duggal et al. 2013b) .

Here we investigated the impact of hip fracture and new onset depressive symptoms on the frequency and function of T_regs_ and CD19^+^ CD24^hi^ CD38^hi^ B cells.

## Materials and methods

### Study participants

101 older hip fracture patients were recruited from five hospitals in Birmingham, UK between 2010 and 2012. Inclusion criteria were that participants had to be aged 60 years and over with a hip fracture sustained 4–6 weeks previously but with no chronic immune-related disorders or taking any regular medications that might modify immunity. Additionally patients must not have had any diagnosis of depression by a physician prior to age 50 years or be taking or have previously taken anti-depressant medication. 43 healthy older adults were also recruited from the community as controls. These controls also had to meet the inclusion criteria above but not have had a hip fracture. The study was approved by South Birmingham Local Research Ethics Committee and all participants provided written informed consent (study ref: 09/H1203/80).

### Study design and procedure

The study was a prospective case–control design with three groups of older adults: hip fracture patients with or without depressive symptoms and healthy older adults. Consent was gained whilst patients were still in hospital. All patients provided a blood sample and completed questionnaires and structured interviews 4-6 weeks after hip fracture. Control participants attended the University, for one-off blood sampling and completed questionnaires. None of the participants had an acute infection at the time of blood sampling.

### Assessment for depressive symptoms

Standard socio-demographic and health behaviour information were taken and all comorbidities and medications, prescription and over-the-counter, were recorded by the interviewer. The psychological status of the participant was assessed by a Geriatric Depression Scale (GDS) (Yesavage et al. 1982). Depression was defined as a GDS score greater than or equal to six. The Hospital Anxiety and Depression Scale (HADS) was also used to measure depression and anxiety (Zigmond and Snaith 1983). Healthy control participants completed the HADS depression sub-scale in order to check that they did not have significant depressive symptoms.

### Foxp3 staining for identification of T_regs_

Peripheral blood mononuclear cells (PBMCs) were isolated by density centrifugation using Ficoll-Paque™ PLUS (GE Healthcare, Sweden). Isolated PBMCs were resuspended in phosphate buffered saline (PBS) at a concentration of 1 × 10^6^/ml and were stained with anti-human CD3-PEcy7 (eBiosciences, UK; clone: UCHT1), anti-human CD4 Alexa fluor 450 (eBiosciences, UK; clone: RPA-T4) and anti-human CD25 APC (Biolegend, UK; clone: BC96) antibodies for 20 min in the dark at 4 °C. Post incubation, cells were washed with PBS and re-suspended in Foxp3 Fix Perm Working solution (eBiosciences, UK) and incubated for 30 min in the dark at room temperature. Post incubation, cells were washed and resuspended in Foxp3 Permeabilization buffer (eBiosciences, UK) and stained with anti-human Foxp3 PE antibody (eBiosciences, UK) for 30 min in the dark at room temperature. Finally, the cells were washed and resuspended in PBS for flow cytometric analysis using a Cyan ™ ADP (Dako Ltd, UK). The percentage of CD3^+^ CD4^+^ CD25^+^ Foxp3^+^ T cells were recorded.

### Stimulation of PBMCs to induce IL10 production by CD4 T cells

Cell cultures were performed in RPMI 1640 (Sigma Aldrich, UK) containing 10 % FCS (Biosera, UK) supplemented with glutamine/penicillin/streptomycin (Life Technologies, UK). PBMCs (1 × 10^6^/ml) were stimulated for 4 h with PMA (50 ng/ml; Sigma Aldrich, UK) and Ionomycin (500 ng/ml; Sigma Aldrich, UK) in the presence of Brefeldin A (10 μg/ml; Sigma Aldrich, UK). Post stimulation, cells were washed twice with PBS and stained using anti-human CD3 PEcy7 (eBiosciences, UK; clone: UCHT1) and anti-human CD4 Alexa fluor 450 (eBiosciences, UK; clone: RPA-T4) for 20 min in the dark at 4 °C. Cells were washed and fixed with Reagent A (Fix and Perm kit, Invitrogen, UK) for 30 min in the dark at room temperature. Post incubation, cells were washed and re-suspended in Reagent B (Fix and Perm kit, Invitrogen, UK) and anti-human Alexa fluor 647 IL10 antibody (clone: JES3-9D7) was added to cells that were incubated in the dark at room temperature for 30 min. After washing the cells were resuspended in PBS and analysed on a Cyan ™ ADP (Dako Ltd, UK) and the percentage of CD3^+^ CD4^+^ IL10^+^ T cells and IL10 expression levels (MFI value) by CD4 T cells was recorded.

### Identification of immunosuppressive CD19^+^CD24^hi^CD38^hi^ B cells

Isolated PBMCs (1 × 10^6^/ml) were stained with a combination of fluorochrome conjugated antibodies including; CD19-PE (eBiosciences, clone: HIB19), CD24-FITC (eBiosciences, clone: eBioSN3), CD38-PEcy7 (eBiosciences, clone: HIT2), antibodies for 20 min in dark at 4 °C. Post incubation, the cells were washed and resuspended in PBS for flow cytometric analysis using Cyan ™ ADP (Dako Ltd, UK). The percentage of CD24^hi^ CD38^hi^ CD19^+^ B cells was recorded.

### CD3 stimulation of PBMCs to induce IL10 production by CD19^+^CD24^hi^CD38^hi^ B cells

96 well microtitre plates with round bottom wells (Sarstedt Ltd, Leicester, UK) were coated with anti-CD3 mAb (BD Biosciences) at a concentration of 0.5 μg/ml for 1 h at 37 °C. Isolated PBMCs were plated at 25 × 10^4^ cells/well for 72 h at 37 °C in a humidified atmosphere of 5 % CO_2_ and Brefeldin A (10 μg/ml; Sigma-Aldrich) was added during the last 6 h of the incubation. After culturing cells with stimulus they were washed and stained for a combination of extracellular surface markers to identify IL10 producing CD24^hi^ CD38^hi^ CD19^+^ B cells. Post immunostaining, cells were fixed, permeabilised and stained with anti-human Alexa fluor 647 IL10 antibody (described in “Stimulation of PBMCs to induce IL10 production by CD4 T cells” section). Post immunostaining, cells were washed and resuspended in PBS and analysed on a Cyan ™ ADP (Dako Ltd, UK). The percentage of IL10^+^ CD24^hi^ CD38^hi^ CD19^+^ B cells and IL10 expression levels (MFI value) by CD24^hi^ CD38^hi^ CD19^+^ B cells was recorded.

### T cell independent (LPS) stimulation of PBMCs to induce IL10 production by CD19^+^CD24^hi^CD38^hi^ B cells

Isolated PBMCs were plated at 25 × 10^4^ cells/well in a 96 well microtitre plates with round bottom wells (Sarstedt Ltd, UK) in the presence of LPS isolated from *Escherichia coli* serotype 0111:B4 (1 µg/ml; Peprotech, UK) for 72 h at 37 °C in a humidified atmosphere of 5 % CO_2_. Brefeldin A (10 μg/ml; Sigma-Aldrich, UK) was added during the last 6 h of the incubation. After culturing cells with stimulus they were washed and stained for a combination of extracellular surface markers to identify IL10 producing CD24^hi^ CD38^hi^ CD19^+^ B cells. Post immunostaining, cells were fixed, permeabilised and stained with anti-human Alexa fluor 647 IL10 antibody (described in “Stimulation of PBMCs to induce IL10 production by CD4 T cells” section). Post immunostaining, cells were washed and resuspended in PBS and analysed on a Cyan ™ ADP (Dako Ltd, UK). The percentage of IL10^+^ CD24^hi^ CD38^hi^ CD19^+^ B cells and IL10 expression levels (MFI value) by CD24^hi^ CD38^hi^ CD19^+^ B cells was recorded.

### Serum cortisol assay

Serum cortisol levels were measured by ELISA using a commercial kit (IBL international, Hamburg, Germany) according to manufacturer’s instructions. Intra assay coefficients of variation (CV %) were 6.7.

### Statistical analysis

Univariate ANOVA with least significant difference post hoc tests were used to assess differences between the three groups (hip fracture with depressive symptoms, hip fracture without depressive symptoms and healthy controls). Where demographic variables differed significantly between the three groups analyses were rerun adjusting for these variables using ANCOVA. Pearson’s correlations were used to examine associations between depression score, serum cortisol levels and T and B cell phenotype and IL10 production.

## Results

The demographic details of the study participants have been reported in full previously (Phillips et al. 2013).

### Regulatory T cells in hip fracture patients

**Fig. 1:**
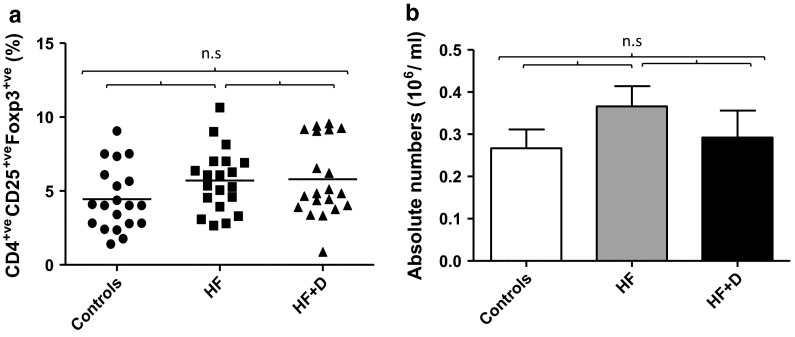
CD4^+^ CD25^+^ Foxp3^+^ regulatory T lymphocytes in hip fracture patients. **a** Percentage of CD4^+^ CD25^+^ Foxp3^+^ T cells in healthy controls (n = 20), hip fracture patients without depressive symptoms (HF; n = 20) and hip fracture patients with depressive symptoms (HF + D; n = 20). The *solid bar* represents the mean value. **b** Absolute number of CD4^+^ CD25^+^ Foxp3^+^ regulatory T cells in healthy controls (n = 12), hip fracture patients without depressive symptoms (HF; n = 13) and hip fracture patients with depressive symptoms (HF + D; n = 10). Data are mean ± SEM

T_regs_ have been defined by the expression of CD4, CD25 and the transcription factor foxp3 (Buckner 2010; O’Garra and Vieira 2004). On examining the circulating frequency of CD4^+^ CD25^+^ Foxp3^+^ T cells no significant differences were observed between hip fracture patients with and without depressive symptoms and healthy older adults, F(2, 57) = 1.24, p = .29, η^2^ = .04 (Fig. 1a). Similarly, no significant differences were found between absolute numbers of regulatory T cells, F (2, 31) = .98, p = .38, η^2^ = 06 (Fig. 1b) between our three subject groups.

### IL10 production by CD4 T cells in hip fracture patients

**Fig. 2:**
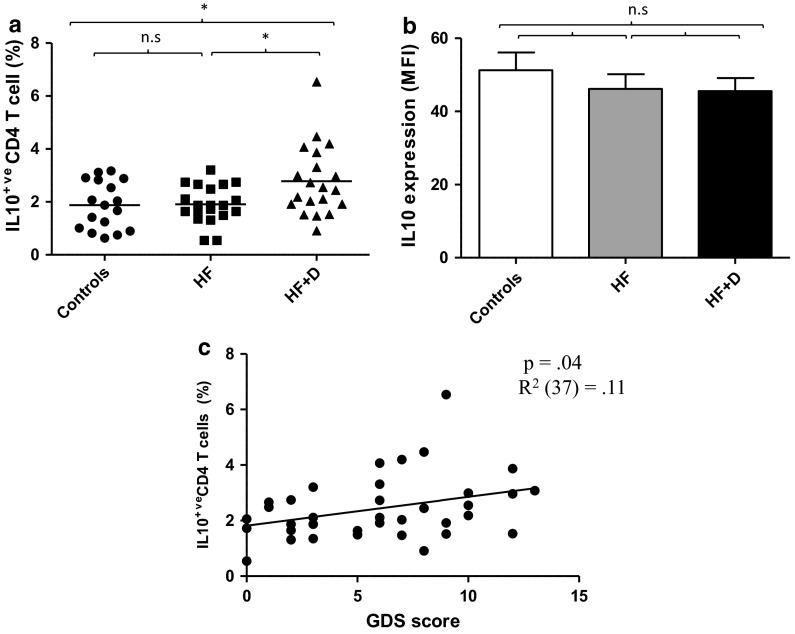
IL10 production by CD4 T lymphocytes in hip fracture patients.** a** Percentage IL10^+^ CD4 T cells and **b** mean IL10 production (MFI value) by CD4 T cells in healthy controls (n = 17), hip fracture patients without depressive symptoms (HF; n = 16) or hip fracture patients with depressive symptoms (HF+D; n = 21). The *solid bar* represents the mean value in (**a**) and data are mean ± SEM in (**b**). **c** Correlation between GDS depression scores and IL10 production by CD4 T cells in hip fracture patients (n = 36). *p < 0.05

IL10 secretion is one of the main mechanisms of immunosuppression used by T_regs_ (Roncarolo et al. 2006). In this study, IL10 production by CD4 T cells upon stimulation with PMA and Ionomycin was measured. Significant differences were seen in the percentage of IL10 producing CD4 T cells between our three groups, F (2, 50) = 4.93, p = .01, η^2^ = .16 (Fig. 2a), driven by a significant increase in the percentage of IL10^+^ CD4 T cells in hip fracture patients with depressive symptoms compared with hip fracture patients without depressive symptoms, p = .04 and healthy controls, p = .02. No significant differences were observed in the amount of IL10 expressed (MFI value) by CD4 T cells between our groups, F(2, 42) = .46, p = .63, η^2^ = .02 (Fig. 2b). Interestingly, there was a significant association between GDS scores and the frequency of peripheral IL10^+^ CD4 T cells in hip fracture patients, β = .34, p = .04, ΔR^2^ = .11, such that hip fracture patients with greater depressive symptoms (GDS score) had a higher frequency of IL10^+^ CD4 T cells (Fig. 2c).

### Frequency of CD19^+^ CD24^hi^ CD38^hi^ B cells in hip fracture patients

**Fig. 3:**
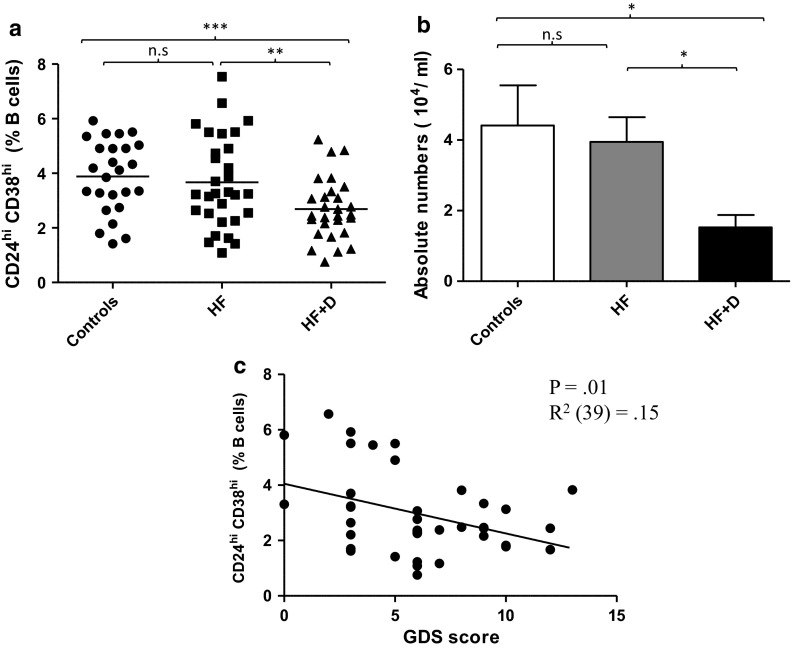
Frequency of CD19^+^CD24^hi^CD38^hi^ B cells in hip fracture patients.** a** Percentage and **b** absolute numbers of CD19^+^CD24^hi^CD38^hi^ cells in healthy controls (n = 21), hip fracture patients without depressive symptoms (HF; n = 15) and hip fracture patients with depressive symptoms (HF + D; n = 22). The *solid bar* represents the mean value in (**a**) and the data are mean ± SEM in (**b**). **c** Correlation between percentage of CD19^+^CD24^hi^CD38^hi^ and GDS scores in hip fracture patients (n = 36). *p < 0.05, **p < 0.005 and ***p < 0.001

Three major populations of circulating B cells have been reported in peripheral blood: transitional, circulating mature naïve and memory B cells discriminated on the basis of relative distribution of developmentally regulated markers CD24 and CD38 (Sims et al. 2005). The gating strategy used to identify CD19^+^ CD24^hi^ CD38^hi^ B cells has been reported previously (Duggal et al. 2013b). On assessing the peripheral frequency and absolute numbers of B cells no differences were observed between hip fracture patients with and without depression and healthy controls (data not shown), which is in agreement with other reports of unaltered circulating B cell numbers in depressed individuals (Basterzi et al. 2010). However the frequency of CD19^+^CD24^hi^CD38^hi^ B cells in the B cell pool differed between our three groups, F(2, 61) = 11.16, p < .001, η^2^ = .26 (Fig. 3a), due to a decline in this subset in hip fracture patients with depressive symptoms compared with healthy controls, p < .001 as well as hip fracture patients without depressive symptoms, p = .001. When all of the above analyses were repeated with adjustment for age, sex and BMI, the results still remained significant (data not shown).

Similarly, significant differences were seen in absolute numbers of CD19^+^CD24^hi^CD38^hi^ B cells, F (2, 38) = 4.85, p = .01, η^2^ = .20 (Fig. 3b) between our three groups, driven by a significant decline in absolute numbers in hip fracture patients with depressive symptoms compared with healthy controls, p = .04 as well as hip fracture patients without depressive symptoms, p = .02. A significant association was observed between GDS scores and frequency of peripheral CD19^+^CD24^hi^CD38^hi^ B cells in hip fracture patients, β = −0.38, p = 0.01, ΔR^2^ = 0.15, such that hip fracture patients with greater depressive symptoms (GDS score) had lower frequency of CD19^+^CD24^hi^CD38^hi^ B cells (Fig. 3c).

### IL10 production by CD19^+^ CD24^hi^ CD38^hi^ B cells in hip fracture patients

**Fig. 4:**
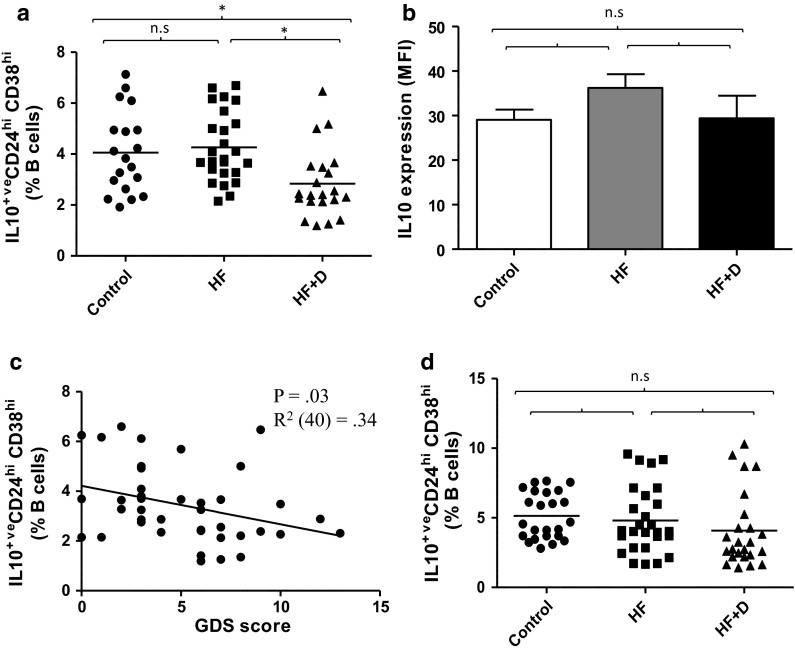
IL10 production by CD19^+^CD24^hi^CD38^hi^ B cells in hip fracture patients.** a** Frequency of IL10^+^CD19^+^CD24^hi^CD38^hi^ cells in healthy controls (n = 18), hip fracture patients without depressive symptoms (HF; n = 21) or hip fracture patients with depressive symptoms (HF+D; n = 19). The *solid bar* represents the mean value. **b** IL10 production by CD19^+^CD24^hi^CD38^hi^ cells in healthy controls (n = 18), hip fracture patients without depressive symptoms (HF; n = 21) or hip fracture patients with depressive symptoms (HF+D; n = 19) post CD3 stimulation for 72 h. Data are mean ± SEM. **c** Correlation between percentage of IL10^+^CD19^+^CD24^hi^CD38^hi^ B cells post CD3 stimulation (72 h) and GDS scores in hip fracture patients (n = 40). **d** IL10 production by CD19^+^CD24^hi^CD38^hi^ cells in healthy controls (n = 24), hip fracture patients without depressive symptoms (HF; n = 26) or hip fracture patients with depressive symptoms (HF+D; n = 22) post LPS stimulation for 72 h. *p < 0.05

The hallmark of CD19^+^ CD24^hi^ CD38^hi^ B cell suppressive function is their ability to produce IL10 (Vitale et al. 2010). CD40 is a co-stimulatory molecule known to be involved in T cell mediated B cell activation (Castigli et al. 1994) known to upregulate IL10 production (Mauri et al. 2003). We have previously reported that CD19^+^ CD24^hi^ CD38^hi^ B cells are the main IL10 producing B cell subset post CD40 stimulation (Duggal et al. 2013b). Thus, the frequency of IL10 producing CD19^+^CD24^hi^CD38^hi^ B cells post CD3 stimulation (72 h) was determined. The data revealed significant differences between our groups, F (2, 55) = 4.88, p = .01, η^2^ = .15, but the reduction in IL10 induction was restricted to hip fracture patients with depressive symptoms compared with healthy controls, p = .04 and hip fracture patients without depression, p = .01 (Fig. 4a). When all of the above analyses were repeated with adjustment for age, sex and BMI, the results still remained significant (data not shown). However, the mean IL10 production by CD19^+^CD24^hi^CD38^hi^ B cells post stimulation did not differ between the subject groups, F(2, 55) = 0.85, p = 0.43, η^2^ = 0.03 (Fig. 4b).

There was a significant association between GDS scores and the frequency of IL10 producing CD19^+^CD24^hi^CD38^hi^ B cells post stimulation in hip fracture patients, β = −0.34, p = .03, ΔR^2^ = 0.34, such that hip fracture patients with greater depressive symptoms (GDS score) had a lower frequency of CD19^+^CD24^hi^CD38^hi^ B cells (Fig. 4c).

In addition to T cell-dependent stimulation, B cells can also be activated by microbial products via Toll-like receptor (TLR) signalling (Yanaba et al. 2009). In a previous study we showed that CD19^+^ CD24^hi^ CD38^hi^ B cells are the main IL10 producing B cell subset post TLR4 ligand (LPS) stimulation (Duggal et al 2013b). On examining IL10 production by CD19^+^ CD24^hi^ CD38^hi^ B cells on stimulation by LPS no significant differences were observed between our groups, F(2, 41) = 2.03, p = .14, η^2^ = .43 (Fig. 4d), although a trend towards a decline in IL10 production by B_regs_ was observed in hip fracture patients with depressive symptoms.

### Serum cortisol and regulatory immune cell parameters

**Fig. 5:**
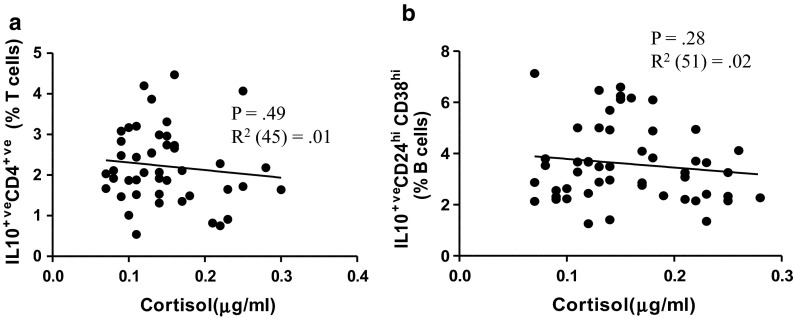
Association between serum cortisol and regulatory cell frequency. The frequency of IL10 producing **a** CD4 T cells and **b** CD19^+^CD24^hi^CD38^hi^ B cells in patients 6 weeks after hip fracture was correlated with the serum cortisol level

We have previously reported elevated serum cortisol levels in the hip fracture patients with depressive symptoms compared with patients with hip fracture alone and healthy controls []. However, there was no significant association between serum cortisol levels and IL10 producing CD4 T cells in hip fracture patients, β = −0.10, p = 0.49, ΔR^2^ = 0.02 (Fig. 5a). Further, there was no significant association between serum cortisol levels and IL10 producing CD19^+^CD24^hi^CD38^hi^ B cells in hip fracture patients, β = −0.15, p = 0.28, ΔR^2^ = 0.02 (Fig. 5b). When all of the above analyses were repeated with adjustment for age and BMI, the results remained the same.

## Discussion

A high incidence of depression has been observed after hip fracture by many studies and was seen in approximately one-third of our hip fracture patients at 6 weeks post-surgery, which is consistent with previous studies (Holmes and House 2000; Lenze et al. 2007). Although mood disorders, especially depression, have been well studied in older patients with fractured neck of femur (Holmes and House 2000; Lenze et al. 2007), the effects of depressive symptoms on immune functioning are less well studied or appreciated in the clinical context. We have recently published that neutrophil bactericidal properties (Duggal et al. 2013a), monocyte (Duggal et al. 2014) and NK cell functioning (Duggal et al. 2015) are impaired only in those hip fracture patients that developed depressive symptoms. We have also shown that new onset of depressive symptoms is associated with poor physical recovery after hip fracture (Phillips et al. 2013). Here we have now shown the effects of chronic stress (hip fracture and depression) on two regulatory immune cell subsets; CD4^+^CD25^+^Foxp3^+^ (T_regs_) and CD19^+^CD24^hi^CD38^hi^ (B_regs_) and again find that depressive symptoms are the factor determining the immune alterations.

Firstly, on examining the effect of hip fracture and depressive symptoms on T_regs_, no significant differences were observed in the frequency of circulating CD4^+^CD25^+^Foxp3^+^ T_regs_, which contradicts the findings of two previous studies showing a decline in T_regs_ in mice with depression-like behaviour (Kim et al. 2012) and in humans with major depression (Li et al. 2010). Furthermore, another study has shown that anti-depressant therapy increased T_reg_ numbers and this was associated with a reduction in depression and reduced serum pro-inflammatory cytokines which have a role in the pathophysiology of depression (Himmerich et al. 2010). One possible explanation of this contradiction might be heterogeneity in T_reg_ characterisation, the three previous studies classified CD4^+^ CD25^+^ T cells as T_regs_, rather than the more accurate CD4^+^ CD25^+^ Foxp3^+^ phenotype and many of these cells would have been CD25 expressing activated T cells rather than Foxp3 expressing immunosuppressive T_regs_. The regulatory function of T_regs_ is known to be mediated via immunosuppressive cytokines such as IL10 (Vignali et al. 2008). IL10 is an immunoregulatory cytokine that plays a central role in controlling inflammation by suppressing T cell proliferation and cytokine production, antigen presentation and proinflammatory cytokine production by monocytes (Moore et al. 2001). We have previously reported higher circulating levels of IL10 in hip fracture patients with new onset of depression (22.41 ± 6.46 pg/ml) compared with hip fracture patients without depression (10.37 ± 2.25 pg/ml) or healthy controls (5.27 ± 1.11 pg/ml) (Duggal et al. 2013a) and here we show a marked increase in IL10 production by CD4 T cells of hip fracture patients with depressive symptoms compared to healthy controls. These data suggest that this is likely to account for the raised IL10 seen in these patients and may contribute to the reduced function of a variety of immune cells seen after hip fracture. Interestingly, a significant positive association was also found between GDS scores and IL10 production by CD4 T cells. Although this is the first study to examine the effect of hip fracture and depression on IL10 production by CD4^+^ T cells, a few studies examining the effect of chronic stress such as caregiving (Damjanovic et al. 2007; Glaser et al. 2001) and academic stress (Koh et al. 2008) have reported increased IL10 production and secretion. Whether the raised IL10 production is a response to the inflammation associated with depression in our cohort, we found raised IL6 and TNFα only in the hip fracture patients who developed depressive symptoms (Duggal et al. 2013a), remains to be shown but is one possibility. HPA axis hyperactivity is one of the most recognised findings in depressed patients (Gold et al. 1988). In-vitro studies have shown that glucocorticoids enhance IL10 production by CD4 T cells (Franchimont 2004; Richards et al. 2000). We have previously reported elevated serum cortisol levels in hip fracture patients with depressive symptoms (Duggal et al. 2013a). However, we failed to find any association between frequency of IL10^+^ CD4 T cells and serum cortisol levels in hip fracture patients.

Recently B_regs_ have attracted increasing attention for the important role of these cells in maintaining immune homeostasis. Studies on B_regs_ in humans are still limited and our study is the first to examine the effects of ageing and chronic stress on these immunosuppressive cells. We have previously reported a numerical deficit in CD19^+^CD24^hi^CD38^hi^ B cells with advancing age. In this study on examining the additive effect of chronic stress on this age associated numerical deficit we report a further reduction in the frequency of B_regs_ (CD19^+^CD24^hi^CD38^hi^) in hip fracture patients with depressive symptoms. In addition we also found impairments in immunosuppressive properties of these cells with age (Duggal et al. 2013b) and here we show an additional decline in IL10 production upon CD3 stimulation in hip fracture patients with depressive symptoms. Again to the best of our knowledge this is the first study to examine the effect of psychological stress on IL10 production by B cells. However, these data are surprising when considered together with the increased IL10 production by CD4 T cells shown here and the overall raised serum IL10 reported in these patients previously (Duggal et al. 2013a). Although the effect of glucocorticoids on B_regs_ remains unexplored, we failed to find any association between frequencies of IL10^+^ B_regs_ and serum cortisol levels in hip fracture patients.

From these data we have to conclude that the increased frequency and function of the T_reg_ population dominates in these patients and contributes to the reduced function seen in a variety of immune cells in these patients (Duggal et al. 2013a, 2015).

## Conclusion

In conclusion, the present study reports for the first time that development of new onset depressive symptoms in older hip fracture patients results in numerical and functional deficits in B_regs_ but an increase in IL10 production by CD4 T cells. These findings suggest that impairments in T regulatory immune cells might be a contributing factor towards immune suppression in hip fracture patients with depressive symptoms and our data support the need for early prevention and treatment of depression in older hip fracture patients.
